# Therapeutic options in peripheral T cell lymphoma

**DOI:** 10.1186/s13045-016-0267-0

**Published:** 2016-04-12

**Authors:** Yaping Zhang, Wei Xu, Hong Liu, Jianyong Li

**Affiliations:** Department of Hematology, The First Affiliated Hospital of Nanjing Medical University, Jiangsu Province Hospital, Nanjing, 210029 China; Department of Hematology, Affiliated Hospital of Nantong University, Nantong, 226000 China; Collaborative Innovation Center For Cancer Personalized Medicine, Nanjing Medical University, Nanjing, 210029 China

**Keywords:** Therapy, Novel target, Peripheral T cell lymphoma

## Abstract

Peripheral T cell lymphoma (PTCL) is a rare and heterogeneous group of non-Hodgkin lymphomas with a very poor prognosis. The standard first-line treatments have resulted in unsatisfactory patient outcomes. With the exception of low-risk anaplastic lymphoma kinase (ALK)-positive anaplastic large cell lymphoma (ALCL), the majority of patients relapse rapidly; the current 5-year overall survival rates are only 10–30 %. Novel targeted therapies and combination chemotherapies are required for the treatment of patients with PTCL. In recent years, some retrospective and prospective studies have been performed concerning PTCL. Consequently, a number of novel agents and their relevant combination therapies have been identified, including histone deacetylase inhibitors, immunoconjugates, antifolates, monoclonal antibodies, immunomodulatory agents, nucleoside analogs, proteasome inhibitors, kinase inhibitors, bendamustine, l-asparaginase, and other targeted agents. It is hoped that these innovative approaches will finally improve outcomes in patients with PTCL. This review summarizes the currently available approaches for the treatment of PTCL with an emphasis on potential new agents, including the role of stem cell transplantation.

## Background

Peripheral T cell lymphoma (PTCL) is a rare and heterogeneous group of clinically aggressive diseases associated with poor prognosis, which represents 10–15 % of non-Hodgkin lymphomas. Twenty-three subtypes of PTCL have been identified. They have been classified into four groups using the World Health Organization (WHO) classification system 2008, based on their clinical features as follows: nodal, extranodal, cutaneous, and leukemic [1]. The International PTCL project that collected 1314 cases of T/NK-cell lymphomas from 22 institutions worldwide revealed that the most common subtypes worldwide are the nodal T cell lymphomas [2]. In the nodal T cell lymphomas, the major subtypes are PTCL, not otherwise specified (PTCL-NOS), angioimmunoblastic T cell lymphoma (AITL), anaplastic lymphoma kinase (ALK)-positive anaplastic large cell lymphoma (ALCL) and ALK-negative ALCL. Among them, PTCL-NOS has been reported as the major subtype worldwide and typically represents a variant that does not meet the criteria for other subtypes.

The treatment approach of PTCL has traditionally been similar to diffuse large B cell lymphoma (DLBCL); however, outcomes are poor when PTCL is treated according to strategies established for aggressive B cell lymphomas [3]. So far, the standard first-line therapy still consists of cyclophosphamide, doxorubicin, vincristine, and prednisone (CHOP) or a CHOP-like regimen. Therapeutic responses to this approach have been neither adequate nor durable [4, 5]. Because of poor outcome, there is an urgent need to optimize induction therapy by incorporating novel agents that target the different pathways. Novel therapeutic options that have been evaluated include histone deacetylase (HDAC) inhibitors (HDACi), pralatrexate, and monoclonal antibodies [6]. This review summarizes the currently available approaches to treat PTCL, including the role of stem cell transplantation (SCT).

### Conventional therapy

Normally, CHOP is still the preferred choice of frontline treatment for PTCL, although it has been disappointing with the exception of low-risk ALK-positive ALCL. A systematic review evaluated CHOP or CHOP-like regimens in 2815 patients with PTCL, with a 5-year overall survival (OS) of 38.5 % [4]. The German high-grade non-Hodgkin’s Lymphoma study group (DSHNHL) analyzed 343 patients with 289 having tumors that belonged to one of the four major subtypes of PTCL. Treatment consisted of six to eight courses of CHOP or etoposide plus (CHOEP). It was noted that the ALK-positive ALCL patients did particularly well with CHOEP, with a significant improvement in both event-free survival and OS. Older patients (age, > 60 years) did worse with the addition of etoposide [5]. Because patients with ALK-positive ALCL responded impressively to CHOP or CHOEP, the National Comprehensive Cancer Network (NCCN) guidelines recommend the CHOP-21 or CHOEP-21 regimens as the first-line therapy in ALK-positive ALCL. Because of less favorable results, NCCN still recommends clinical trials for first-line therapy in all other subtypes [7]. Several trials have investigated whether the efficacy of CHOP could be increased by the addition of novel agents as first-line therapy; this is discussed below.

### Novel agents and relevant combination therapies

To improve the outcome of patients with PTCL, the introduction of novel agents is necessary. Recent research has led to the development of numerous agents, including HDACi, immunoconjugates, antifolates, monoclonal antibodies, immunomodulatory agents, nucleoside analogs, proteasome inhibitors, kinase inhibitors, bendamustine, l-asparaginase, and other targeted agents. Some of these novel agents can also be combined with other therapies to enhance their efficacy.

#### Histone deacetylase inhibitors

There are approximately 18 different HDACs, which are overexpressed in several tumor types. HDAC1 expression is significantly higher in PTCL, suggesting a probable mechanism of tumor suppression and sensitivity to HDACi in T cell lymphoma (TCL) [8]. HDACi induces histone acetylation, leading to increased expression of tumor suppressor genes, consequently resulting in cell-cycle arrest and cell differentiation. Two drugs have been approved by the US Food and Drug Administration (FDA) for PTCL: romidepsin and belinostat.

##### Romidepsin

Romidepsin is a potent HDACi with a good efficacy and safety profile in relapsed/refractory (R/R) PTCL. It was approved by the FDA in 2011 for the treatment of PTCL in patients who had received ≥ 1 prior therapies. A previous phase II clinical trial (NCT00007345) evaluated patients with refractory PTCL who had been treated with romidepsin (14 mg/m^2^ administered as a 4-h infusion on days 1, 8, and 15 of a 28-day cycle). There were 45 patients included in this investigation who had a median of three prior treatments. The overall response rate (ORR) was 38 %, and eight patients experienced complete remission (CR) after therapy. The median duration of response (DoR) was 8.9 (range 2–74) months. The most common (≥ 40 %) adverse effects (AEs) included nausea (51 %), thrombocytopenia (47 %), leukopenia (47 %), granulocytopenia (45 %), fatigue (40 %), and anemia (40 %) [9]. In a subsequent pivotal phase II trial, 130 patients with histologically confirmed R/R PTCL were treated with romidepsin at the same dose for six cycles; patients with at least stable disease (SD) were allowed to continue until disease progression (NCT00426764) [10–13]. Fifty patients (38 %) were treated for ≥ 4 cycles, and 36 patients (28 %) received > 6 cycles. Most patients had PTCL-NOS (*n* = 69), AITL (*n* = 27), or ALK-negative ALCL (*n* = 21). The ORR was relatively high (25 %) considering that these patients had failed ≥ 1 prior therapies; 19 patients (15 %) had CR/unconfirmed CR (CRu), ten of whom had a long-term (≥ 12 months) response. The median DoR was 28 months. In patients who achieved CR/CRu, median progression-free survival (PFS) was 29 months and OS was not reached. The most common grade ≥ 3 AEs were thrombocytopenia (24 %), neutropenia (20 %), and infections (19 %). This study also demonstrated that prolonged romidepsin provides clinical benefits for R/R patients who had achieved at least stable disease. Romidepsin has been recommended by the NCCN as a second-line and subsequent therapy in patients, regardless of intention to proceed to high-dose therapy or SCT [7].

Because romidepsin alone has limited efficacy, trials examining the combination of romidepsin with traditional chemotherapy or newer agents are ongoing. In a single-arm, phase Ib/II study (EudraCT2010-020962-91 and NCT01280526), carried out at nine Lymphoma Study Association centers in France, 37 patients with previously untreated PTCL received eight 3-week cycles of CHOP with varying doses of romidepsin (8, 10, or 12 mg/m^2^ on days 1 and 8). Based on this study, the recommended dose of romidepsin for the phase II study was 12 mg/m^2^. Among 35 evaluable patients in this study, the ORR was 69 %, including 51 % with a CR. At the median follow-up of 30 months, the estimated PFS and OS were 41 and 71 %, respectively. Three patients had early cardiac events, including two myocardial infarctions and one acute cardiac failure. No deaths were attributable to toxicity. Twenty-five patients had at least one serious AE; the most common AEs were febrile neutropenia, thrombocytopenia, lung infection, and vomiting [14]. An ongoing phase III study, combining romidepsin with CHOP (RoCHOP), will compare the outcome with CHOP alone, to evaluate patient response rates and survival (NCT01796002). Romidepsin is also currently being investigated in patients with PTCL in various combinations, including with ICE (NCT01590732), lenalidomide (NCT01742793, NCT02232516), gemcitabine (NCT01822886), pralatrexate (NCT01947140), oral 5-azacitidine (NCT01998035), or CHOEP (NCT02223208).

##### Belinostat

Belinostat is a pan-HDAC inhibitor with antitumor and anti-angiogenic properties. Belinostat has demonstrated clinical benefit for patients with R/R PTCL in the BELIEF trial. In this trial, there were 129 patients with R/R PTCL who received belinostat (1000 mg/m^2^ administered on days 1–5 of every 3-week cycle) for a median of two cycles (range 1–33). The majority of patients received CHOP/CHOP-like regimens prior to enrollment (96 %), and the median number of systemic therapies was two. A smaller proportion (23 %) of patients had previously undergone SCT prior to enrollment. Among the 120 evaluable patients, 26 % of the remaining patients demonstrated an ORR, with 11 % having a CR and 15 % a partial response (PR). SD was seen in 15 % while 40 % had progressive disease (PD). In terms of the three most common subtypes, ORR was seen in 23 % of patients with PTCL-NOS, 46 % in AITL, and 15 % in ALK-negative ALCL. The median PFS was 1.6 months, and the median OS was 7.9 months. In addition, 63.3 % of patients exhibited decreased tumor volume, and belinostat treatment enabled 12 patients to undergo SCT. These responses were observed across PTCL subtypes and were durable, with a median DoR of 13.6 months and an ongoing response > 36 months in one patient. The most common grade 3/4 AEs were thrombocytopenia (13 %), neutropenia (13 %), anemia (10 %), dyspnea (6 %), pneumonia (6 %), and fatigue (5 %). Patients mainly discontinued treatment as a result of PD (64 %); 7 % of patients discontinued treatment as a result of AEs [15, 16]. These findings demonstrated the antitumor activity of belinostat, resulting in its FDA approval in 2014 for patients with R/R PTCL; findings also suggested that the combination of belinostat with other therapeutic agents may improve clinical outcomes in PTCL. In another phase II study (NCT00274651), Francine et al. evaluated the efficacy and safety of belinostat (1000 mg/m^2^ administered on days 1–5 of every 3-week cycle) in patients with either PTCL or cutaneous T cell lymphoma (CTCL) [17]. There were 24 patients with PTCL included in this analysis; all had received a median of three prior systemic therapies (range 1–9) and 40 % had stage IV disease. The ORRs were 25 %, including a CR of 8 %. The median DoR was 109 days. In the PTCL cohort, grade 3/4 AEs reported as being related to belinostat included five grade 3 AEs (ileus paralytic, pneumonitis, rash maculo-papular, rash macular, and cellulitis) and one grade 4 AE (thrombocytopenia). Belinostat had no clear clinically relevant effect on electrocardiograms in this trial except for a minimal change in the QTc duration in the 5–10-ms range, which is of minimal clinical importance [17].

##### Chidamide

Chidamide, a new oral isotype-selective HDACi, approved in China for the treatment of R/R PTCL in December 2014, is the first listed benzamide class of HDACi in the world [18]. In a pivotal phase II study (ChiCTR-TNC-10000811), 83 patients with R/R PTCL were treated with chidamide at a dose of 30 mg orally twice per week, and 79 patients was eligible. The ORR was 28 % (22 of 79) including 14 % (11 of 79) with a CR/CRu. The median DoR was 9.9 (range 1.1–40.8) months. The median PFS and OS were 2.1 and 21.4 months, respectively. AITL patients tended to have a higher ORR (50 %) and CR/CRu rate (40 %), as well as more durable responses. Most AEs were grade 1 or 2, and AEs of grade ≥ 3 that occurred in ≥ 10 % patients were thrombocytopenia (22 %), leucopenia (13 %), and neutropenia (11 %), respectively. Chidamide has exhibited significant single-agent activity and manageable toxicity in R/R PTCL in China. As a result of this study, the CFDA has approved chidamide for the treatment of R/R PTCL [19].

#### Immunoconjugates

Fusion proteins have been engineered to achieve the following: act as decoy receptors; increase the deliverability of an active substance into the cells; add stability and recruit immune effector cells; and fulfill other functions. Brentuximab vedotin (BV) is an immunoconjugate that combines an antitubulin agent with a CD30-specific chimeric monoclonal antibody. A phase II study of BV (1.8 mg/kg administered intravenously over 30 min every 3 weeks) in 58 ALCL patients reported an ORR of 86 %, including 57 % with CRs, and a median DoR of 12.6 months. The median OS was not reached at the time of the analysis; the estimated 12-month survival rate was 70 %. The median PFS among all patients was 13.3 months. The most common (≥ 30 %) AEs were peripheral sensory neuropathy (41 %), nausea (40 %), fatigue (38 %), and pyrexia (34 %). The AEs led to treatment discontinuation in 24 % of patients. Based on the results of this study, the US FDA and European Medicines Agency (EMA), in 2011 and 2012, respectively, approved the use of BV for patients with systemic ALCL after failure of ≥ 1 multi-agent chemotherapy regimen (NCT00866047) [20]. Another phase II trial of BV administered as the same dose in 22 PTCL-NOS and 13 AITL patients reported an ORR of 41 % with a median PFS of 2.6 months (NCT01421667). No correlation between CD30 expression per central review and response was observed. Safety data included grade ≥ 3 events involving neutropenia (14 %), peripheral sensory neuropathy (9 %), and hyperkalemia (9 %) [21]. A phase I open-label study evaluated the efficacy and safety of BV at a dose of 1.8 mg/kg every 3 weeks, administered sequentially with CHOP or in combination with CHP (CHOP without vincristine) as frontline treatment in patients with CD30-positive PTCL (NCT01309789). Thirty-nine patients were enrolled in the study (13 and 26 patients in the sequential- and combination-treatment groups, respectively). After sequential treatment, 11 (85 %) patients achieved an objective response (62 % CR; 77 % estimated 1-year PFS), with grade 3/4 AEs in 62 %. At the end of combination treatment, patients (*n* = 26) achieved an objective response (88 % CR; 71 % estimated 1-year PFS). Grade 3/4 AEs (≥ 10 %) in the combination-treatment group were febrile neutropenia (31 %), neutropenia (23 %), anemia (15 %), and pulmonary embolism (12 %). This study demonstrated a manageable safety profile and exhibited substantial antitumor activity in newly diagnosed patients with CD30-positive PTCL [22]. A randomized phase III trial is underway, comparing a combination of BV and CHP with CHOP alone (NCT01777152).

#### Antifolates

Pralatrexate, an antimetabolite drug, selectively enters cells through reduced folate carrier type 1 (RFC-1), which is a protein that transports reduced natural folates into highly proliferative cells. A large prospective study (PROPEL) enrolled 115 patients, of whom 109 were evaluable. Patients received pralatrexate intravenously at a dose of 30 mg/m^2^/week for 6 weeks in 7-week cycles until disease progression or the development of unacceptable toxicity. An ORR of 29 % was reported, with 12 (11 %) patients achieving a CR/CRu, 20 (18 %) a PR, and 21 (19 %) SD. The median DoR was 10.1 months, and the median PFS and OS were 3.5 and 14.5 months, respectively. The most common AEs were mucositis (71 %), and grade 3/4 AEs were thrombocytopenia (33 %), mucositis (22 %), neutropenia (22 %), and anemia (18 %). No cumulative myelosuppression was observed with continued pralatrexate treatment [23]. Based on this study, the US FDA Office of Oncology Drug Products granted accelerated approval to pralatrexate injection for the treatment of patients with R/R PTCL in September 2009. However, in January 2012, the Committee for Medicinal Products for Human Use (CHMP) refused marketing authorization for the FDA-approved pralatrexate for PTCL treatment and confirmed their refusal on 19 April 2012. The CHMP was concerned that this study was designed in a way that did not allow the Committee to assess the benefits of the drug; this was particularly true because pralatrexate was not compared with any other treatment or placebo in another group of patients. Moreover, there was no clear improvement seen in the condition of the patients because the study investigated patient response to treatment but did not further allow the Committee to assess its effect on OS or PFS. The CHMP was of the opinion that there was insufficient evidence to establish the benefits of pralatrexate in the treatment of PTCL. Therefore, the CHMP was of the opinion that the benefits of pralatrexate did not outweigh its risks and recommended that it be refused marketing authorization. Currently, there are only three ongoing clinical trials involving pralatrexate, combined with CHOP (NCT02594267), romidepsin (NCT01947140), or oral leucovorin (NCT02106650).

#### Monoclonal antibodies

##### Alemtuzumab

Alemtuzumab is an anti-CD52 monoclonal antibody that is known to suppress immunity, including the depletion of CD4 and CD8 T cells as well as B cells. It has undergone early stage evaluation, but severe immunocompromise and neutropenia have limited its use. Alemtuzumab combined with other therapies has also been investigated. In a prospective phase II trial, 29 (70.7 %) of the 41 patients received CHO(E)P followed by alemtuzumab consolidation (133 mg in total). The CR rate was 58.5 %, and EFS and OS at 3 years in the whole intent-to-treat population were 32.3 and 62.5 %, respectively; EFS and OS at 3 years were 42.4 and 75.1 % in the patients who had received alemtuzumab. The main grade 3/4 toxicities were infections and neutropenia with one potential treatment-related death (NCT01806337) [24]. A phase II study of patients with newly diagnosed (*n* = 27) or R/R (*n* = 11) PTCL received a combination of alemtuzumab, fludarabine, cyclophosphamide, and doxorubicin. The ORR was 61 %, with a CR of 39 %. In newly diagnosed patients, the ORR was 63 %, the median OS was 25.9 months, and the PFS was 11.8 months. In R/R patients, the median OS was 6.1 months. The most frequent grade 3/4 toxicities were leukopenia (95 % of patients) and thrombocytopenia (58 %). Cytomegalovirus (CMV) reactivation occurred in 12 patients, but only two had CMV disease. Treatment-related deaths occurred in six newly diagnosed patients and one with R/R disease [25]. Alemtuzumab-based therapy was active in PTCL but was also associated with significant toxicity.

##### Mogamulizumab

Mogamulizumab is a monoclonal antibody targeting CC chemokine receptor 4 (CCR4). Regulatory T cells (Tregs) that overexpress CCR4 in aggressive PTCL impair host antitumor immunity and provide an environment for the tumor to grow. Mogamulizumab depletes CCR4-positive Tregs, potentially evoking antitumor activity. A phase II study of weekly administered mogamulizumab infusions at a dose of 1.0 mg/kg in 27 patients with relapsed, aggressive CCR4-positive TCL showed an ORR of 50 %, including 31 % with a CR. The median PFS was 5.2 months, and the median OS was 13.7 months. The most common (≥ 15 %) grade 3/4 AEs were lymphopenia (74 %), leukocytopenia (30 %), thrombocytopenia (19 %), neutropenia (19 %), and rash (19 %) (NCT00920790) [26]. Another phase II study of mogamulizumab at the same dose in 38 patients, of whom 37 were evaluable with relapsed CCR4-positive PTCL or CTCL, reported an ORR of 35 %, including 14 % with a CR; the median PFS was 3 months. The most common (≥ 15 %) grade 3/4 AEs were lymphocytopenia (73 %) and neutropenia (19 %) (NCT01192984) [27].

##### Zanolimumab

Zanolimumab is a biologically active monoclonal antibody that targets the CD4 antigen present on subsets of PTCL. This antibody was studied in a phase II study, including 21 R/R patients with CD4-positive PTCL (NCT00877656). Weekly zanolimumab infusions at a dose of 980 mg for 12 weeks achieved an ORR of 24 %, and 10 % of patients had a CR. Drug-related grade 3 AEs occurred in lymphocytopenia (10 %), infusion-related AEs (10 %), and arthralgia (5 %), which were well tolerated [28].

#### Immunomodulatory agents

The immunomodulatory drug lenalidomide acts by inhibition of vascular endothelial growth factor (VEGF), activation of natural killer cells and T lymphocytes, and modulation of various cytokines such as tumor necrosis factor-α, interleukin-12, and interferon-γ. They also exert a direct effect on cell-cycle arrest and apoptosis, thus being able to target both neoplastic cells and the tumor microenvironment. Lenalidomide (25 mg orally on days 1–21 of each 28-day cycle) has shown efficacy in patients with R/R PTCL [29–31]. A phase II study involving 23 evaluable patients showed an ORR of 30 % (all PRs). The median PFS and OS were 3.2 and 7.9 months, respectively. AEs were consistent with the known safety profile of lenalidomide. The most common grade 3/4 AEs were thrombocytopenia (42 %), neutropenia (21 %), dyspnea (17 %), febrile neutropenia (17 %), pain (17 %), and pneumonitis (17 %). Study discontinuation was mainly for PD (46 %); four patients (17 %) discontinued treatment as a result of AEs [29]. The multicenter phase II EXPECT trial included 54 patients with R/R PTCL, mostly with AITL (*n* = 26; 48 %) and PTCL-NOS (*n* = 20; 37 %). The ORR was 22 % (11 % CR/CRu). The median PFS and DoR were 2.5 and 3.6 months, respectively. The most common grade 3/4 AEs (≥ 15 %) included thrombocytopenia (20 %), gastrointestinal disorders (17 %), neutropenia (15 %), and infections (15 %) [30]. Recently, a phase II trial in 40 patients (one ineligible) with R/R and untreated PTCL reported an ORR of 26 % (8 % CR). Three patients had SD for five cycles (NCT00322985). The median OS was 12 months, the median PFS was 4 months, and the median DoR was 13 months, including five patients with treatment responses that lasted > 1 year. Among the patients who had R/R PTCL (29 patients), the ORR was 24 %, the median OS was 12 months, the median PFS was 4 months, and the median DoR was 5 months. The most common grade 4 AEs were pain, not otherwise specified (21 %), neutropenia (13 %), muscle weakness (10 %), and dyspnea (10 %); dehydration (10 %) was the most commonly reported grade 3 AE [31]. Because of the limited efficacy of lenalidomide as a single agent in PTCL, the combination therapies of lenalidomide and romidepsin are currently under investigation, including in the NCT02232516 trial involving untreated patients and the NCT01742793 trial involving R/R patients.

#### Nucleoside analogs

Nucleoside analogs are chemotherapeutic agents that primarily inhibit DNA replication and repair. These agents are cytotoxic to both proliferating and non-proliferating cells. The nucleoside analogs include gemcitabine, pentostatin, cladribine, fludarabine, clofarabine, forodesine, and nelarabine. Gemcitabine, cladribine, and fludarabine have shown efficacy in PTCL, and gemcitabine is the most effective pyrimidine nucleoside analog in PTCL; it has been included in the NCCN guidelines as second-line therapy for patients with relapsed PTCL [7] and has been incorporated into several combination chemotherapy regimens. Zinzani et al. reported on the efficacy and safety of 20 pretreated PTCL-NOS patients treated with gemcitabine [32]. All patients had stage III–IV disease. Gemcitabine was given on days 1, 8, and 15 involving a 28-day schedule (1200 mg/m^2^/day) for a total of three to six cycles. Patients had a CR rate of 30 % and a PR rate of 25 %. Among the CR patients, five were in continuous CR with a variable disease-free survival (DFS) (15–60 months). Gemcitabine was generally well tolerated; there was no grade 3/4 hematological toxicity, and all of the responding patients completed the treatment [32]. The first prospective CISL trial investigated the efficacy and tolerability of a salvage GDP regimen for 25 R/R PTCL patients. Treatment consisted of gemcitabine at a dose of 1000 mg/m^2^ intravenously on days 1 and 8, dexamethasone at a dose of 40 mg orally on days 1–4, and cisplatin 70 mg/m^2^ intravenously on day 1, every 21 days. The median number of GDP cycles was four. The ORR was 72 %, including 12 patients with a CR (48 %) and six with a PR (24 %). Four patients proceeded to autologous SCT (ASCT), and three patients finally achieved a CR. The median PFS was 9.3 months with a median follow-up duration of 27.1 months. In a total of 86 cycles of GDP, grade 3 or 4 neutropenia and thrombocytopenia occurred in 16.3 and 12.8 % of the cycles, respectively. Three patients (3.3 %) experienced febrile neutropenia [33]. In these studies, gemcitabine was well tolerated and effective.

#### Proteasome inhibitors

Bortezomib, a proteasome inhibitor, has been well tolerated and active as a single or combinational agent in PTCL patients. In a phase II study in 15 relapsed CTCL or PTCL patients, bortezomib (1.3 mg/m^2^ intravenously on days 1, 4, 8, and 11, every 21 days for a total of six cycles) produced an ORR of 67 %, with two CRs and no grade 4 toxicity [34]. A large multicenter phase II trial involving 46 patients evaluated bortezomib plus CHOP in a frontline setting. Although the ORR was 76 % and the CR was 65 %, the 3-year OS and PFS were 47 and 35 %, respectively, as a result of frequent relapse after remission. The outcome was similar to CHOP alone. Grade 3/4 leucopenia was the most frequent toxicity, whereas neurotoxicity was tolerable (NCT00374699) [35]. Another phase I/II trial involved bortezomib (1.6 mg/m^2^) given concurrently with gemcitabine (800 mg/m^2^) on days 1 and 8 of a 21-day cycle. Of 32 patients, 16 each had R/R PTCL and DLBCL. Among the first 18 patients, 67 % experienced grade 3/4 neutropenia and/or grade 3/4 thrombocytopenia resulting in repeated treatment delays. Thus, the study was amended to give bortezomib and gemcitabine on days 1 and 15 of a 28-day cycle, which resulted in markedly improved tolerability. The ORR for PTCL was 36 % (CR, 27 %). Among six PTCL patients treated using the modified schedule, the intent-to-treat ORR was 50 % (CR, 30 %) [36]. The NCCN has recommended bortezomib as a second-line therapy for patients without intention to proceed to transplantation [7].

#### Kinase inhibitors

##### Aurora A kinase (AAK) inhibitors

AAK is a mitotic kinase implicated in oncogenesis and has been found to be upregulated in PTCL, most strongly in ALK-positive ALCL, followed by ALK-negative ALCL and PTCL-NOS [37]. A phase II trial evaluated its efficacy against a variety of NHL. In this trial, patients received alisertib at a dose of 50 mg twice daily for 7 days in 21-day cycles, until PD or unacceptable toxicities developed. The ORR in 48 enrolled patients was 27 and 50 % in eight PTCL patients. Common drug-related grade 3/4 AEs included neutropenia (63 %), leukopenia (54 %), anemia (35 %), thrombocytopenia (33 %), and stomatitis (15 %) [38]. Another phase II intergroup trial (SWOG 1108) enrolled 37 pretreated PTCL patients. Among the PTCL subtypes, the ORR was 30 %; however, no responses were observed in patients with transformed Mycosis fungoides. The median DoR was 3 months. The estimated median PFS and OS were 3 and 8 months, respectively. Grade 3/4 AEs in ≥ 5 % of patients included neutropenia (32 %), anemia (30 %), thrombocytopenia (24 %), febrile neutropenia (14 %), mucositis (11 %), and rash (5 %). Treatment was most frequently discontinued because of disease progression. Based on these results, a randomized phase III trial of alisertib versus the investigator’s choice is underway for patients with relapsed PTCL (NCT01482962) [39].

##### Tyrosine kinase

In patients with ALK-positive ALCL, there are efforts to study the use of crizotinib, an oral small-molecule tyrosine kinase inhibitor of ALK, which has been FDA approved for the treatment of lung cancer harboring a translocation in the ALK gene. In a small study consisting of 11 patients with refractory ALK-positive lymphoma, nine had ALCL histology. Patients received crizotinib at a dose of 250 mg twice daily as monotherapy until disease progression. There was an ORR in 91 % of patients. The OS was 72.7 %, and the PFS was 63.7 % at 2 years; three patients had a CR for > 30 months under continuous crizotinib administration. All toxicities were grade 1/2, including ocular flashes, peripheral edema, and neutropenia [40]. Crizotinib exerted potent antitumor activity in advanced ALK-positive lymphoma patients; a clinical trial evaluating crizotinib in patients with primary ALK-positive systemic ALCL is ongoing (NCT02487316).

#### Bendamustine

Bendamustine is an alkylating agent with antimetabolite properties that exhibits activity in several hematologic malignancies and solid tumors. In the BENTLY trial, 60 patients with CTCL and PTCL (mainly AITL and PTCL-NOS) were treated with bendamustine infused at a dose of 120 mg/m^2^ on days 1 and 2 every 3 weeks, for six cycles. Twenty patients (33 %) received fewer than three cycles of bendamustine, mostly because of disease progression. The ORR was 50 %, including a CR in 17 patients (28 %) and a PR in 13 patients (22 %). The median DoR was 3.5 months, with 30 % of responses lasting > 6 months. The median PFS and OS were 3.6 and 6.2 months, respectively. The most frequent (≥ 5 %) grade 3/4 AEs were neutropenia (30 %), thrombocytopenia (24 %), and infections (20 %). Infections and hematological AEs led to discontinuation in five patients (8 %) [41]. Because bendamustine showed an encouraging response rate and acceptable toxicity, the NCCN has recommended it as a second-line and subsequent therapy, regardless of high-dose therapy and SCT [7].

#### l-asparaginase

l-asparaginase (L-ASP) is a naturally occurring enzyme and exerts its antitumor activity by depleting circulating pools of L-asparagine, which is an essential amino acid for protein synthesis. Few reports have shown that L-ASP can be effective against PTCL as well as NK/T cell lymphomas. Tsutomu et al. presented a relapse patient with Epstein-Barr virus (EBV)-positive cytotoxic PTCL-NOS, who was successfully treated using only L-ASP. L-ASP treatment was initiated at 6000 U/m^2^ on days 1, 3, 5, 10, and 12 together with prednisolone at a dose of 1 mg/kg. Although he developed grade 2 liver dysfunction and grade 2 coagulopathy, the patient achieved CR status [42]. A retrospective study analyzed 102 patients with incipient PTCL who received L-ASP (administered as 6000 U/m^2^, once a day, for 7 days) with or without multi-drug chemotherapy regimens. Patients who received L-ASP containing multi-drug chemotherapy (L-ASP group) had higher ORRs than those who received non L-ASP (83.3 vs. 61.7 %; *P* = 0.016), particularly those at stage III/IV (82.4 vs. 54 %; *P* = 0.007) and with an international prognostic index (IPI) score of ≥ 2 (82.1 vs. 50 %; *P* = 0.006). The median OS of the L-ASP and non L-ASP groups was 10.5 and 13 months, respectively, and the median PFS was 10 and 11 months, respectively. The 3-year OS rate of the L-ASP and non L-ASP groups was 48.9 and 65 %, respectively, and the 3-year PFS rate was 40.8 and 61 %, respectively. None of these values differed significantly. Although the incidence of AEs was higher in the L-ASP group, most of them were mild and controllable after supportive treatment. There was no significant difference in serious infections caused by grade 3/4 neutropenia between the two groups (*P* = 0.777). Other severe AEs in the L-ASP group, such as hematencephalon and acute pancreatitis, were only seen in one case. L-ASP showed better short-term efficacy in PTCL patients [43].

#### Other targeted agents

Some other novel agents in use or under investigation seem to have more targeted action. The mTOR pathway is constitutively activated in the TCL cells and is responsible for TCL proliferation. A phase II study first demonstrated that mTORC1 inhibitors (everolimus) have substantial antitumor activity in relapsed TCL patients (NCT00436618). In the clinical trial, 16 patients with relapsed TCL were enrolled and received oral everolimus at a dose of 10 mg daily. The ORR was 44 % (7/16). The median PFS was 4.1 months, and the median OS was 10.2 months. The median DoR for the seven responders was 8.5 months. Seven patients had a grade ≥3 hematological toxicity and six had grade 3 nonhematological toxicity [44]. Recent studies have shown that specific inhibition of IDH mutants can reverse the abnormal methylations because of the mutation, and had efficacy in pre-clinical and clinical studies [45]; AG-221 acted on IDH2 mutation and is in a clinical trial (NCT02273739). A recent case study reported on the efficacy of the hypomethylating agent 5-azacytidine in an AITL patient with a TET2 mutation [46]. Other novel therapeutic agents, such as ruxolitinib, that act on the JAK/STAT pathway are being investigated in clinical trials (e.g., NCT01431209). Clinical trial efficacy results regarding novel single-agent treatments, as well as ongoing clinical trials of PTCL, are summarized in Tables 1 and 2.

**Table 1:** Clinical trial efficacy results of novel single-agent treatments

Agent	Targeting site	Phase	No. of evaluable patients	Type of patients	Outcomes	ClinicalTrial.gov ID	References
Tipifarnib	Farnesyltransferase inhibitor	II	12	PTCL	50 % ORR	NCT00082888	[49]
Darinaparsin	Organic arsenic compound	II	7	PTCL	28.6 % ORR, 3.6 months median TTF, 6.4 months OS	NCT00421213	[50]
Panobinostat	Pan-deacetylase inhibitor	II	139	CTCL	17.3 % ORR	NCT00425555	[51]
Denileukin diftitox	Interleukin fusion protein	III	263	CTCL	38 % ORR, median DoR was 277 days	NCT00050999	[52]
						NCT00051012	
Ixazomib	Proteasome inhibitor	I	4	PTCL	1 PR	NCT00893464	[53]
Sorafenib	Multikinase inhibitor	—	12	PTCL/CTCL	42 % ORR, 4 CR, 1 PR	—	[54]
Forodesine	PNP inhibitor	II	101	CTCL	No CR, 11 % PR, 50 % SD, median DoR was 191 days	NCT00501735	[55]

*ORR* overall response rate, *CR* complete response, *PR* partial response, *SD* stable disease, *TTF* time to treatment failure, *OS* overall survival, *DoR* duration of response, *PNP* purine nucleoside phosphorylase

**Table 2:** Ongoing clinical trials of novel agents in patients with PTCL

Agent	Targeting site	Phase	ClinicalTrial.gov ID
Endostar	Angiogenesis inhibitor	II	NCT02520219
Selinexor	Selective inhibitor of nuclear export	II	NCT02314247
Tipifarnib	Farnesyltransferase inhibitor	II	NCT02464228
Darinaparsin	Organic arsenic compound	I	NCT01435863
			NCT01689220
Ixazomib	Proteasome inhibitor	II	NCT02158975
Denileukin diftitox	Interleukin fusion protein	II	NCT00050999
			NCT00051012
Forodesine	PNP inhibitor	I/II	NCT01776411
Ruxolitinib	JAK inhibitor	II	NCT01431209
Temsirolimus	mTOR inhibitor	I	NCT01614197
Carfilzomib	Proteasome inhibitor	I	NCT01336920
Panobinostat	Pan-deacetylase inhibitor	II	NCT01261247
Clofarabine	DNA synthesis inhibitor	I/II	NCT00644189
MK2006	AKT inhibitor	II	NCT01258998
Sorafenib	Multikinase inhibitor	II	NCT00131937
Alefacept	Immunosuppressive dimeric fusion protein	I	NCT00438802
Pembrolizumab	PD-1 antibody	II	NCT02535247

*PNP* purine nucleoside phosphorylase, *JAK* Janus kinases, *mTOR* mammalian target of rapamycin, *PD-1* programmed death-1

### Stem cell transplantation

Relapses in patients with PTCL are common; therefore, SCT as a consolidation and salvage strategy has been chosen to enhance therapeutic results. The NCCN guideline has recommended ASCT as an option for consolidation after first remission in patients with histologies other than ALK-positive ALCL with low international prognostic index scores [7, 47]. However, there have been no randomized controlled trials to restrict patients who can benefit from ASCT or achieve an objective response immediately after induction chemotherapy, with the exception of low-risk ALK-positive ALCL. Moreover, the role of ASCT in relapsed PTCL remains to be determined and thus cannot be compared with allogeneic SCT (allo-SCT). Some limited studies have evaluated the efficacy of ASCT and allo-SCT in relapsed PTCL. The largest retrospective study included 76 patients with TCL who had been treated using ASCT or allo-SCT protocols at the MD Anderson Cancer Center between 1990 and 2009. For ASCT for relapsed disease, 41 patients were studied. The 4-year OS and PFS rates were 50 and 38 %, respectively, for ASCT patients. Patients with CR2/3 had a 4-year OS rate of 59 % after ASCT, suggesting that remission status before transplantation is required for good results. This study also presented the role of allo-SCT in 35 PTCL patients in relapsed disease. With a median follow-up of 45 months for allo-SCT, the 4-year OS and PFS were 36 and 28 %, respectively. The 4-year non-relapse mortality (NRM) rate was significantly higher in allo-SCT than in ASCT patients (40 vs. 17 %; *P* < 0.001) [48]. Allo-SCT did not result in a superior outcome relative to ASCT, and the inferior OS survival seen with allo-SCT was in large part caused by the increased NRM.

## Conclusions

Although the treatment of PTCL has undergone significant changes during the last few years, the response rate and long-term effect for patients with PTCL remains unsatisfactory. An increasing number of studies have identified signal pathway and gene abnormalities in PTCL, such as the NF-κB and mTOR pathways, and the IDH and TET2 mutations. There is no specific stratification of therapy for all PTCLs. Genetic and proteomic techniques probably contribute to the accurate treatment options. For instance, ALK-positive PTCL patients treated with crizotinib or brentuximab vedotin exhibit preferable responses. Multiple pathways are involved in the pathogenesis of some specific subtypes of PTCL, which make combined therapy possible. Some studies involving the combination of romidepsin with pralatrexate or lenalidomide are ongoing. We expect further research results in the future.

As well as novel targeted agents, the role and timing of stem cell transplantation in the majority of PTCL patients are still elusive. This problem is difficult to resolve because of insufficient data from randomized controlled trials involving rare and heterogeneous PTCL. Investigators are faced with the challenge of selecting from the promising strategies available for PTCL subtypes. New diagnostic tools and molecular genetic markers could further subdivide PTCL patients and assist clinical investigators to select the correct therapeutic options. Individualized treatment would be a promising approach for PTCL in the future.

## References

[1] Swerdlow SH, Campo E, Harris NL, Jaffe ES, Pileri SA, Stein H, Thiele J, Vardiman JW (2008). WHO classification of tumours of haematopoietic and lymphoid tissues.

[2] Vose J, Armitage J, Weisenburger D (2008). International peripheral T-cell and natural killer/T-cell lymphoma study: pathology findings and clinical outcomes. J Clin Oncol.

[3] Foss F (2011). Hematology: relapsed and refractory PTCL—into the therapeutic abyss. Nat Rev Clin Oncol.

[4] Abouyabis AN, Shenoy PJ, Sinha R, Flowers CR, Lechowicz MJ (2011). A systematic review and meta-analysis of front-line anthracycline-based chemotherapy regimens for peripheral T-cell lymphoma. ISRN Hematol.

[5] Schmitz N, Trumper L, Ziepert M, Nickelsen M, Ho AD, Metzner B, Peter N, Loeffler M, Rosenwald A, Pfreundschuh M (2010). Treatment and prognosis of mature T-cell and NK-cell lymphoma: an analysis of patients with T-cell lymphoma treated in studies of the German High-Grade Non-Hodgkin Lymphoma Study Group. Blood.

[6] Karlin L, Coiffier B (2014). The changing landscape of peripheral T-cell lymphoma in the era of novel therapies. Semin Hematol.

[7] NCCN Clinical Practice Guidelines in Oncology. Non-Hodgkin’s lymphoma, V.2.2015. http://www.nccn.org/professionals/physician_gls/f_guidelines.asp. Accessed 23 March 2015.

[8] Marquard L, Poulsen CB, Gjerdrum LM, de Nully BP, Christensen IJ, Jensen PB, Sehested M, Johansen P, Ralfkiaer E (2009). Histone deacetylase 1, 2, 6 and acetylated histone H4 in B- and T-cell lymphomas. Histopathology.

[9] Piekarz RL, Frye R, Prince HM, Kirschbaum MH, Zain J, Allen SL, Jaffe ES, Ling A, Turner M, Peer CJ, Figg WD, Steinberg SM, Smith S, Joske D, Lewis I, Hutchins L, Craig M, Fojo AT, Wright JJ, Bates SE (2011). Phase 2 trial of romidepsin in patients with peripheral T-cell lymphoma. Blood.

[10] Coiffier B, Pro B, Prince HM, Foss F, Sokol L, Greenwood M, Caballero D, Borchmann P, Morschhauser F, Wilhelm M, Pinter-Brown L, Padmanabhan S, Shustov A, Nichols J, Carroll S, Balser J, Balser B, Horwitz S (2012). Results from a pivotal, open-label, phase II study of romidepsin in relapsed or refractory peripheral T-cell lymphoma after prior systemic therapy. J Clin Oncol.

[11] Coiffier B, Pro B, Prince HM, Foss F, Sokol L, Greenwood M, Caballero D, Morschhauser F, Wilhelm M, Pinter-Brown L, Padmanabhan Iyer S, Shustov A, Nielsen T, Nichols J, Wolfson J, Balser B, Horwitz S (2014). Romidepsin for the treatment of relapsed/refractory peripheral T-cell lymphoma: pivotal study update demonstrates durable responses. J Hematol Oncol.

[12] Foss F, Coiffier B, Horwitz S, Pro B, Prince HM, Sokol L, Greenwood M, Lerner A, Caballero D, Baran E, Kim E, Nichols J, Balser B, Wolfson J, Whittaker S (2014). Tolerability to romidepsin in patients with relapsed/refractory T-cell lymphoma. Biomark Res.

[13] Foss F, Horwitz S, Pro B, Prince HM, Sokol L, Balser B, Wolfson J, Coiffier B (2016). Romidepsin for the treatment of relapsed/refractory peripheral T cell lymphoma: prolonged stable disease provides clinical benefits for patients in the pivotal trial. J Hematol Oncol.

[14] Dupuis J, Morschhauser F, Ghesquieres H, Tilly H, Casasnovas O, Thieblemont C, Ribrag V, Bossard C, Bras FL, Bachy E, Hivert B, Nicolas-Virelizier E, Jardin F, Bastie JN, Amorim S, Lazarovici J, Martin A, Coiffier B (2015). Combination of romidepsin with cyclophosphamide, doxorubicin, vincristine, and prednisone in previously untreated patients with peripheral T-cell lymphoma: a non-randomised, phase 1b/2 study. Lancet Haematol.

[15] O’Connor OA, Masszi T, Savage KJ (2013). Belinostat, a novel pan-histone deacetylase inhibitor (HDACi), in relapsed or refractory peripheral T-cell lymphoma (R/R PTCL): Results from the BELIEF trial. J Clin Oncol (ASCO Meeting Abstact).

[16] O’Connor OA, Horwitz S, Masszi T, Van Hoof A, Brown P, Doorduijn J, Hess G, Jurczak W, Knoblauch P, Chawla S, Bhat G, Choi MR, Walewski J, Savage K, Foss F, Allen LF, Shustov A (2015). Belinostat in patients with relapsed or refractory peripheral T-cell lymphoma: results of the Pivotal Phase II BELIEF (CLN-19) Study. J Clin Oncol.

[17] Foss F, Advani R, Duvic M, Hymes KB, Intragumtornchai T, Lekhakula A, Shpilberg O, Lerner A, Belt RJ, Jacobsen ED, Laurent G, Ben-Yehuda D, Beylot-Barry M, Hillen U, Knoblauch P, Bhat G, Chawla S, Allen LF, Pohlman B (2015). A Phase II trial of Belinostat (PXD101) in patients with relapsed or refractory peripheral or cutaneous T-cell lymphoma. Br J Haematol.

[18] Gu R, Liu T, Zhu X, Gan H, Wu Z, Li J, Zheng Y, Dou G, Meng Z (2015). Development and validation of a sensitive HPLC-MS/MS method for determination of chidamide (epidaza), a new benzamide class of selective histone deacetylase inhibitor, in human plasma and its clinical application. J Chromatogr B Analyt Technol Biomed Life Sci.

[19] Shi Y, Dong M, Hong X, Zhang W, Feng J, Zhu J, Yu L, Ke X, Huang H, Shen Z, Fan Y, Li W, Zhao X, Qi J, Zhou D, Ning Z, Lu X (2015). Results from a multicenter, open-label, pivotal phase II study of chidamide in relapsed or refractory peripheral T-cell lymphoma. Ann Oncol.

[20] Pro B, Advani R, Brice P, Bartlett NL, Rosenblatt JD, Illidge T, Matous J, Ramchandren R, Fanale M, Connors JM, Yang Y, Sievers EL, Kennedy DA, Shustov A (2012). Brentuximab vedotin (SGN-35) in patients with relapsed or refractory systemic anaplastic large-cell lymphoma: results of a phase II study. J Clin Oncol.

[21] Horwitz SM, Advani RH, Bartlett NL, Jacobsen ED, Sharman JP, O’Connor OA, Siddiqi T, Kennedy DA, Oki Y (2014). Objective responses in relapsed T-cell lymphomas with single-agent brentuximab vedotin. Blood.

[22] Fanale MA, Horwitz SM, Forero-Torres A, Bartlett NL, Advani RH, Pro B, Chen RW, Davies A, Illidge T, Huebner D, Kennedy DA, Shustov AR (2014). Brentuximab vedotin in the front-line treatment of patients with CD30+ peripheral T-cell lymphomas: results of a phase I study. J Clin Oncol.

[23] O’Connor OA, Pro B, Pinter-Brown L, Bartlett N, Popplewell L, Coiffier B, Lechowicz MJ, Savage KJ, Shustov AR, Gisselbrecht C, Jacobsen E, Zinzani PL, Furman R, Goy A, Haioun C, Crump M, Zain JM, Hsi E, Boyd A, Horwitz S (2011). Pralatrexate in patients with relapsed or refractory peripheral T-cell lymphoma: results from the pivotal PROPEL study. J Clin Oncol.

[24] Binder C, Ziepert M, Pfreundschuh M, Duhrsen U, Eimermacher H, Aldaoud A, Rosenwald A, Loeffler M, Schmitz N, Truemper L (2013). CHO(E)P-14 followed by alemtuzumab consolidation in untreated peripheral T cell lymphomas: final analysis of a prospective phase II trial. Ann Hematol.

[25] Weidmann E, Hess G, Chow KU, Krause SW, Subklewe M, Kruse J, Weisel KC, Soekler M, Kim SZ, Napieralski S, Rech J, Dreyling M, Jager E, Mitrou PS (2010). A phase II study of alemtuzumab, fludarabine, cyclophosphamide, and doxorubicin (Campath-FCD) in peripheral T-cell lymphomas. Leuk Lymphoma.

[26] Ishida T, Joh T, Uike N, Yamamoto K, Utsunomiya A, Yoshida S, Saburi Y, Miyamoto T, Takemoto S, Suzushima H, Tsukasaki K, Nosaka K, Fujiwara H, Ishitsuka K, Inagaki H, Ogura M, Akinaga S, Tomonaga M, Tobinai K, Ueda R (2012). Defucosylated anti-CCR4 monoclonal antibody (KW-0761) for relapsed adult T-cell leukemia-lymphoma: a multicenter phase II study. J Clin Oncol.

[27] Ogura M, Ishida T, Hatake K, Taniwaki M, Ando K, Tobinai K, Fujimoto K, Yamamoto K, Miyamoto T, Uike N, Tanimoto M, Tsukasaki K, Ishizawa K, Suzumiya J, Inagaki H, Tamura K, Akinaga S, Tomonaga M, Ueda R (2014). Multicenter phase II study of mogamulizumab (KW-0761), a defucosylated anti-cc chemokine receptor 4 antibody, in patients with relapsed peripheral T-cell lymphoma and cutaneous T-cell lymphoma. J Clin Oncol.

[28] d’Amore F, Radford J, Relander T, Jerkeman M, Tilly H, Osterborg A, Morschhauser F, Gramatzki M, Dreyling M, Bang B, Hagberg H (2010). Phase II trial of zanolimumab (HuMax-CD4) in relapsed or refractory non-cutaneous peripheral T cell lymphoma. Br J Haematol.

[29] Dueck G, Chua N, Prasad A, Finch D, Stewart D, White D, van der Jagt R, Johnston J, Belch A, Reiman T (2010). Interim report of a phase 2 clinical trial of lenalidomide for T-cell non-Hodgkin lymphoma. Cancer.

[30] Morschhauser F, Fitoussi O, Haioun C, Thieblemont C, Quach H, Delarue R, Glaisner S, Gabarre J, Bosly A, Lister J, Li J, Coiffier B (2013). A phase 2, multicentre, single-arm, open-label study to evaluate the safety and efficacy of single-agent lenalidomide (Revlimid) in subjects with relapsed or refractory peripheral T-cell non-Hodgkin lymphoma: the EXPECT trial. Eur J Cancer.

[31] Toumishey E, Prasad A, Dueck G, Chua N, Finch D, Johnston J, van der Jagt R, Stewart D, White D, Belch A, Reiman T (2015). Final report of a phase 2 clinical trial of lenalidomide monotherapy for patients with T-cell lymphoma. Cancer.

[32] Zinzani PL, Venturini F, Stefoni V, Fina M, Pellegrini C, Derenzini E, Gandolfi L, Broccoli A, Argnani L, Quirini F, Pileri S, Baccarani M (2010). Gemcitabine as single agent in pretreated T-cell lymphoma patients: evaluation of the long-term outcome. Ann Oncol.

[33] Park BB, Kim WS, Suh C, Shin DY, Kim JA, Kim HG, Lee WS (2015). Salvage chemotherapy of gemcitabine, dexamethasone, and cisplatin (GDP) for patients with relapsed or refractory peripheral T-cell lymphomas: a consortium for improving survival of lymphoma (CISL) trial. Ann Hematol.

[34] Zinzani PL, Musuraca G, Tani M, Stefoni V, Marchi E, Fina M, Pellegrini C, Alinari L, Derenzini E, de Vivo A, Sabattini E, Pileri S, Baccarani M (2007). Phase II trial of proteasome inhibitor bortezomib in patients with relapsed or refractory cutaneous T-cell lymphoma. J Clin Oncol.

[35] Kim SJ, Yoon DH, Kang HJ, Kim JS, Park SK, Kim HJ, Lee J, Ryoo BY, Ko YH, Huh J, Yang WI, Kim HK, Min SK, Lee SS, Do IG, Suh C, Kim WS (2012). Bortezomib in combination with CHOP as first-line treatment for patients with stage III/IV peripheral T-cell lymphomas: a multicentre, single-arm, phase 2 trial. Eur J Cancer.

[36] Evens AM, Rosen ST, Helenowski I, Kline J, Larsen A, Colvin J, Winter JN, van Besien KM, Gordon LI, Smith SM (2013). A phase I/II trial of bortezomib combined concurrently with gemcitabine for relapsed or refractory DLBCL and peripheral T-cell lymphomas. Br J Haematol.

[37] Kanagal-Shamanna R, Lehman NL, O’Donnell JP, Lim MS, Schultz DS, Chitale DA, Bueso-Ramos CE, Medeiros LJ, Inamdar KV (2013). Differential expression of aurora-A kinase in T-cell lymphomas. Mod Pathol.

[38] Friedberg JW, Mahadevan D, Cebula E, Persky D, Lossos I, Agarwal AB, Jung J, Burack R, Zhou X, Leonard EJ, Fingert H, Danaee H, Bernstein SH (2014). Phase II study of alisertib, a selective Aurora A kinase inhibitor, in relapsed and refractory aggressive B- and T-cell non-Hodgkin lymphomas. J Clin Oncol.

[39] Barr PM, Li H, Spier C, Mahadevan D, LeBlanc M, Ul Haq M, Huber BD, Flowers CR, Wagner-Johnston ND, Horwitz SM, Fisher RI, Cheson BD, Smith SM, Kahl BS, Bartlett NL, Friedberg JW (2015). Phase II intergroup trial of alisertib in relapsed and refractory peripheral T-cell lymphoma and transformed mycosis fungoides: SWOG 1108. J Clin Oncol.

[40] Gambacorti Passerini C, Farina F, Stasia A, Redaelli S, Ceccon M, Mologni L, Messa C, Guerra L, Giudici G, Sala E, Mussolin L, Deeren D, King MH, Steurer M, Ordemann R, Cohen AM, Grube M, Bernard L, Chiriano G, Antolini L, Piazza R (2014). Crizotinib in advanced, chemoresistant anaplastic lymphoma kinase-positive lymphoma patients. J Natl Cancer Inst.

[41] Damaj G, Gressin R, Bouabdallah K, Cartron G, Choufi B, Gyan E, Banos A, Jaccard A, Park S, Tournilhac O, Schiano-de Collela JM, Voillat L, Joly B, Le Gouill S, Saad A, Cony-Makhoul P, Vilque JP, Sanhes L, Schmidt-Tanguy A, Bubenheim M, Houot R, Diouf M, Marolleau JP, Bene MC, Martin A, Lamy T (2013). Results from a prospective, open-label, phase II trial of bendamustine in refractory or relapsed T-cell lymphomas: the BENTLY trial. J Clin Oncol.

[42] Takahashi T, Ikejiri F, Onishi C, Kawakami K, Inoue M, Miyake T, Tanaka J, Araki A, Maruyama R, Ohshima K, Suzumiya J (2010). L-asparaginase-induced complete response in a relapsed patient with Epstein-Barr virus and cytotoxic peripheral T-cell lymphoma not otherwise specified. Intern Med.

[43] Yao G, Zhou D, Zhou M, Bao C, He D, Li L, Zhu J, He J, Shi J, Zheng W, Cai Z, Huang H, Ye X, Xie W (2015). Clinical analysis and prognostic significance of L-asparaginase containing multidrug chemotherapy regimen in incipient peripheral T-cell lymphoma. Int J Clin Exp Med.

[44] Witzig TE, Reeder C, Han JJ, LaPlant B, Stenson M, Tun HW, Macon W, Ansell SM, Habermann TM, Inwards DJ, Micallef IN, Johnston PB, Porrata LF, Colgan JP, Markovic S, Nowakowski GS, Gupta M (2015). The mTORC1 inhibitor everolimus has antitumor activity in vitro and produces tumor responses in patients with relapsed T-cell lymphoma. Blood.

[45] Wang C, McKeithan TW, Gong Q, Zhang W, Bouska A, Rosenwald A, Gascoyne RD, Wu X, Wang J, Muhammad Z, Jiang B, Rohr J, Cannon A, Steidl C, Fu K, Li Y, Hung S, Weisenburger DD, Greiner TC, Smith L, Ott G, Rogan EG, Staudt LM, Vose J, Iqbal J, Chan WC (2015). IDH2R172 mutations define a unique subgroup of patients with angioimmunoblastic T-cell lymphoma. Blood.

[46] Cheminant M, Bruneau J, Kosmider O, Lefrere F, Delarue R, Gaulard P, Radford I, Derrieux C, Hermine O, Lemonnier F (2015). Efficacy of 5-azacytidine in a TET2 mutated angioimmunoblastic T cell lymphoma. Br J Haematol.

[47] d’Amore F, Relander T, Lauritzsen GF, Jantunen E, Hagberg H, Anderson H, Holte H, Osterborg A, Merup M, Brown P, Kuittinen O, Erlanson M, Ostenstad B, Fagerli UM, Gadeberg OV, Sundstrom C, Delabie J, Ralfkiaer E, Vornanen M, Toldbod HE (2012). Up-front autologous stem-cell transplantation in peripheral T-cell lymphoma: NLG-T-01. J Clin Oncol.

[48] Beitinjaneh A, Saliba RM, Medeiros LJ, Turturro F, Rondon G, Korbling M, Fayad L, Fanale MA, Alousi AM, Anderlini P, Betul O, Popat UR, Pro B, Khouri IF (2015). Comparison of survival in patients with T cell lymphoma after autologous and allogeneic stem cell transplantation as a frontline strategy or in relapsed disease. Biol Blood Marrow Transplant.

[49] Witzig TE, Tang H, Micallef IN, Ansell SM, Link BK, Inwards DJ, Porrata LF, Johnston PB, Colgan JP, Markovic SN, Nowakowski GS, Thompson CA, Allmer C, Maurer MJ, Gupta M, Weiner G, Hohl R, Kurtin PJ, Ding H, Loegering D, Schneider P, Peterson K, Habermann TM, Kaufmann SH (2011). Multi-institutional phase 2 study of the farnesyltransferase inhibitor tipifarnib (R115777) in patients with relapsed and refractory lymphomas. Blood.

[50] Hosein PJ, Craig MD, Tallman MS, Boccia RV, Hamilton BL, Lewis JJ, Lossos IS (2012). A multicenter phase II study of darinaparsin in relapsed or refractory Hodgkin’s and non-Hodgkin’s lymphoma. Am J Hematol.

[51] Duvic M, Dummer R, Becker JC, Poulalhon N, Ortiz Romero P, Grazia Bernengo M, Lebbe C, Assaf C, Squier M, Williams D, Marshood M, Tai F, Prince HM (2013). Panobinostat activity in both bexarotene-exposed and -naive patients with refractory cutaneous T-cell lymphoma: results of a phase II trial. Eur J Cancer.

[52] Duvic M, Geskin L, Prince HM (2013). Duration of response in cutaneous T-cell lymphoma patients treated with denileukin diftitox: results from 3 phase III studies. Clin Lymphoma Myeloma Leuk.

[53] Assouline SE, Chang J, Cheson BD, Rifkin R, Hamburg S, Reyes R, Hui AM, Yu J, Gupta N, Di Bacco A, Shou Y, Martin P (2014). Phase 1 dose-escalation study of IV ixazomib, an investigational proteasome inhibitor, in patients with relapsed/refractory lymphoma. Blood Cancer J.

[54] Gibson JF, Foss F, Cooper D, Seropian S, Irizarry D, Barbarotta L, Lansigan F (2014). Pilot study of sorafenib in relapsed or refractory peripheral and cutaneous T-cell lymphoma. Br J Haematol.

[55] Dummer R, Duvic M, Scarisbrick J, Olsen EA, Rozati S, Eggmann N, Goldinger SM, Hutchinson K, Geskin L, Illidge TM, Giuliano E, Elder J, Kim YH (2014). Final results of a multicenter phase II study of the purine nucleoside phosphorylase (PNP) inhibitor forodesine in patients with advanced cutaneous T-cell lymphomas (CTCL) (Mycosis fungoides and Sezary syndrome). Ann Oncol.

